# Plasma Levels of mir-34a-5p Correlate with Systemic Inflammation and Low Naïve CD4 T Cells in Common Variable Immunodeficiency

**DOI:** 10.1007/s10875-023-01618-0

**Published:** 2023-12-22

**Authors:** Sofia Nyström, Jonas Hultberg, Emelie Blixt, Åsa Nilsdotter-Augustinsson, Marie Larsson

**Affiliations:** 1 Department of Clinical Immunology and Transfusion Medicine, and Department of Biomedical and Clinical Sciences, Linköping University, S-58185 Linköping, Sweden; 2 Division of Molecular Medicine and Virology, Department of Biomedical and Clinical Sciences, Linköping University, Linköping, Sweden; 3 Division of Inflammation and Infection, Department of Biomedical and Clinical Sciences, Linköping University, Linköping, Sweden

**Keywords:** Inborn errors of immunity, common variable immunodeficiency, immune dysregulation, circulating miRNAs, next generation sequencing

## Abstract

**Purpose:**

Common variable immunodeficiency (CVID) is a primary antibody deficiency that commonly manifests as recurrent infections. Many CVID patients also suffer from immune dysregulation, an inflammatory condition characterized by polyclonal lymphocytic tissue infiltration and associated with increased morbidity and mortality. The genetic cause is unknown in most CVID patients and epigenetic alterations may contribute to the broad range of clinical manifestations. MicroRNAs are small non-coding RNAs that are involved in epigenetic modulation and may contribute to the clinical phenotype in CVID.

**Methods:**

Here, we determined the circulating microRNAome and plasma inflammatory proteins of a cohort of CVID patients with various levels of immune dysregulation and compared them to healthy controls. A set of deregulated microRNAs was validated by qPCR and correlated to inflammatory proteins and clinical findings.

**Results:**

Levels of microRNA-34a correlated with 11 proteins such as CXCL9, TNF, and IL10, which were predicted to be biologically connected. Moreover, there was a negative correlation between mir-34 levels and the number of naïve CD4 T cells in CVID.

**Conclusion:**

Collectively, our data show that microRNAs correlate with the inflammatory response in CVID. Further investigations are needed to elucidate the role of miRNAs in the development of CVID-related immune dysregulation.

**Supplementary Information:**

The online version contains supplementary material available at 10.1007/s10875-023-01618-0.

## Introduction

Common variable immunodeficiency disorder (CVID) is an inborn error of immunity with an estimated incidence of 1:20000 in Europe. It is a heterogeneous disorder characterized by hypogammaglobinemia [1–3]. Immunoglobulin replacement therapy reduces the number of bacterial infections [4]. Many CVID patients develop non-infectious complications that relate to immune dysregulation (ImD) and contribute to increased morbidity and mortality [4]. CVID-related ImD includes autoimmunity, lymphocytic infiltration of non-lymphoid tissues, lymphoid hyperplasia, and lymphoma [2]. Patients with increased frequencies of atypical B cells with low expression of CD21 and decreased naïve CD4 T cells are at higher risk of ImD complications [5–7]. CVID-related ImD is associated with increased interferon (IFN)-γ activity [8–10]. Treatment to mitigate ImD in CVID is so far mostly restricted to nonspecific immunosuppressive therapy [11, 12].

Currently, pathogenic monogenic variants have been identified in 35% of the patients with CVID [13, 14]. Variants of genes originally identified in non-CVID immunodeficiencies have also been observed in CVID patients, emphasizing the complex underlying genetics of this disorder [15, 16]. Alterations in epigenetic regulators can cause malfunctions of the immune system [17]. In addition to genetic variants, epigenetic changes may contribute to the development of CVID [16, 18]. It was recently reported that epigenetic alterations affecting DNA methylation may contribute to B cell failure in CVID [19].

MicroRNAs (miRNA) are short non-coding RNAs that are involved in epigenetic regulation and function mainly to repress gene expression. For instance, miRNAs are involved in the regulation of B cell differentiation and have been associated with autoimmune disease and B cell lymphomas [20–23]. Increased T cell expression of mir-201 has been reported in a small cohort of CVID patients [24]. Changes in circulating miRNAs reflect ongoing biological processes in the tissues and can be used as biomarkers in cancer, autoimmune, and infectious diseases [25–27]. Alteration of miRNA signatures in CVID can be considered the result of epigenetic processes and may contribute to the pathophysiology of CVID and CVID-related ImD. Epigenetic studies on CVID are scarce.

In the present study, we used next-generation sequencing to profile plasma miRNA signatures in CVID patients with and without ImD. Selected miRNAs were validated by polymerase chain reaction (PCR) and correlated to plasma proteins and the levels of naïve CD4 T cells. Predicting disease-associated miRNAs may increase the understanding of the disease mechanisms of CVID and pave the way for new treatment strategies.

## Methods

### Ethical Approval

This study was approved by the Regional Ethics Committee of Linköping, Sweden (2017/214–32). All study subjects enrolled in this study gave their written informed consent to participate in this study.

### Study Subjects

The definition of CVID in this study follows the former Swedish Guidelines on Primary Immunodeficiency Disorders; in brief, serum IgG < 3.0g/L and IgA < 0.07g/L in the absence of any secondary immune deficiency. The cohort included 10 CVID patients with no to moderate ImD, *i.e.,* infections only (CVID_InO_), and 10 CVID patients with severe ImD complications (CVID_C_). Clinical data were retrieved from their medical records and covered the time of the study start and 10 years back. An evaluation document was used to score the burden of infections and ImD as previously described [10]. In brief, patients were assessed for the presence of ImD across three items: autoimmunity, gastroenteropathy and lymphoproliferation. Each item was scored 0–4; hence, the sum formed an ImD score between 0 and 12. Patients were also given an infection score between 0 and 4 based on the number of suspected bacterial infections per year after initiation of IgG-substitution therapy. Information about B cell subsets was retrieved from the clinical records, and patients were classified according to the Freiburg CVID criteria [28]. Patients were compared to a group of healthy controls recruited among blood donors, who were matched for sex and age (Table 1).

**Table 1:** Demographics and clinical characteristics of study participants

	CVID_InO_ (*n* = 10)	CVID_C_ (*n* = 10)	HC (*n* = 10)
Age, years, median (range)	57 (21–76)	51 (22–74)	52 (28–68)
Sex, female/male	1/9	4/6	4/6
CVID duration, years, median (range)	> 10 (0 to > 10)	6 (1 to > 10)	N/A
Freiburg class 1 (1a),* n*	5 (2)	9 (8)	N/A
CD4^+^ T cells/µL, median (range)	491 (338–1260)	739 (289–1766)	811 (274–1743)
CD8^+^ T cells/µL, median (range)	482 (225–1126)	474 (187–2328)	372 (160–703)
CD4/CD8, median (range)	1.4 (0.6–2.2)	1.7 (0.2–2.6)	2.2 (1.2–4.3)

*InO*, infection only; *C*, CVID with immune dysregulation complications; *HC*, healthy controls

### Materials

A detailed list of all key resources including reagents for flow cytometry, critical commercial assays, software, and algorithms is available in supplementary information Table S1.

### Blood Sample Collection and Plasma Preparation

Blood samples were obtained in vacutainer tubes (BD Biosciences). The samples were kept at room temperature for up to 5 h before the plasma was separated and stored at − 80°C until used.

### Flow Cytometry for Quantification of Peripheral T Cells

Absolute T cell numbers were determined by flow cytometry with the use of Tru count tubes (BD Biosciences). Briefly, antibodies were added to whole blood and samples were lysed with FACS lysing solution (BD biosciences) before data were acquired using FACS Canto II (BD Biosciences). Kaluza flow cytometry software version 1.5 (Beckman Coulter) was used for data analysis.

### RNA Extraction, Library Construction, and High-Throughput Sequencing

RNA extraction, library construction, and high-throughput analyses were carried out by QIAGEN Genomic Services in Hilden, Germany. In brief, RNA extracted from plasma with miRNeasy kit (QIAGEN) was converted into miRNA next-generation sequencing libraries. Adapters containing unique molecular identifiers (UMI) were ligated to the RNA. Then, the RNA was converted to cDNA and amplified using PCR. Libraries were pooled in equimolar ratios and quantified by qPCR before being sequenced on a NextSeq (Illumina Inc., San Diego, CA, USA) according to the manufacturer’s instructions.

### The miRNA-seq Data Analysis

Raw data was de-multiplexed and FASTQ files for each sample were generated using the bcl2fastq software (Illumina Inc.). All primary analysis was carried out using CLC Genomics Server 21.0.4 (Qiagen). The workflow “QIAseq miRNA Quantification” of the CLC Genomics Server with standard parameters was used to map the reads to miRBase version 22. In short, the reads were trimmed, before filtering of reads with length < 15 nt or length > 55 nt. The reads were then deduplicated using their UMI. Reads were grouped based on the UMI sequences. Reads that failed mapping to the miRbase were mapped to the human genome, using the “RNA-Seq Analysis” workflow of the CLC Genomics Server.

### Identification of Differentially Expressed miRNAs

The empirical analysis of the differential gene expression algorithm of the CLC Genomics Workbench was used for differential expression analysis. For all unsupervised analysis, only miRNAs that had at least 10 counts summed over all samples were considered. A variance stabilizing transformation was performed on the raw count matrix using the function of the R package DESeq2 version 1.28.1. Five hundred genes with the highest variance were used for the principal component analysis. Thirty-five genes with the highest variance across samples were selected for hierarchical clustering.

### Targeted Analysis of miRNA with Real-Time qPCR

In brief, extracted RNA was reverse transcribed using the miRCURY LNA RT Kit (QIAGEN). cDNA was assayed in PCR reactions according to the protocol for miRCURY LNA miRNA PCR using miRCURY LNA SYBR Green master mix. The amplification was performed in a LightCycler® 480 Real-Time PCR System (Roche Diagnostics, Basel, Switzerland) in 384 well plates. Target miRNA Cq values were normalized to global mean Cq of hsa-miR-23a-3p and hsa-let-7d-3p, using the formula:

$$\mathrm{Normalized}\;\mathrm{dCq}=\mathrm{global}\;\mathrm{mean}\;\mathrm{Cq}\;\left(\mathrm{sample}\;n\right)-\mathrm{target}\;\mathrm{Cq}\;\left(\mathrm{sample}\;n\right)$$

### Target Gene Prediction

The miRNA Enrichment Analysis and Annotation Tool (miEAA) was used for target gene prediction using over-representation analysis (ORA) with at least three hits among input deregulated miRNAs and FDR < 0.01. When CVID_C_- or CVID_InO_ groups were compared to healthy controls, differently expressed miRNAs with FDR < 0.01 were considered deregulated and miRNAs with FDR < 0.1 when the CVID_C_ group was compared to the CVID_InO_ group. The Uniprot database was used to find Biological Process GO-terms of predicted target genes.

### Targeted Proteomic Analysis

Frozen plasma samples were sent to the Clinical Biomarkers facility, Science for Life Laboratory, Uppsala, Sweden, for analysis. The multiplex protein extension assay (PEA), OLINK Target 96 Inflammation v.3021, was used for the detection of 92 different inflammatory-associated proteins (Table S2). Seventy-two proteins were detected in > 95% of samples and selected for further analysis. Thirty-two of these proteins were differently expressed (*t*-test FDR < 0.05) when comparing CVID patients with healthy controls and were used for the construction of a correlation matrix with normalized miRNA dCq-values.

### Protein–Protein Interaction Analysis

Protein–protein interaction networks were created with differently expressed proteins using STRING version 11.5 (http://string-db.org/) by including interactions with a medium confidence score (> 0.7). STRING database is a curated knowledge database of known and predicted protein–protein interactions [29].

### Statistical Analysis

An Exact Test was used for comparing miRNA profiles generated by high-throughput sequencing, and Benjamini–Hochberg correction was applied to adjust for multiple testing. The Mann–Whitney test and Kruskal–Wallis test with Dunn’s correction were used for univariate statistics comparing two or three groups, respectively. Spearman’s was used for correlation measures.

## Results

### Demographics and Clinical Characteristics of the Patient Cohort

**Fig. 1:**
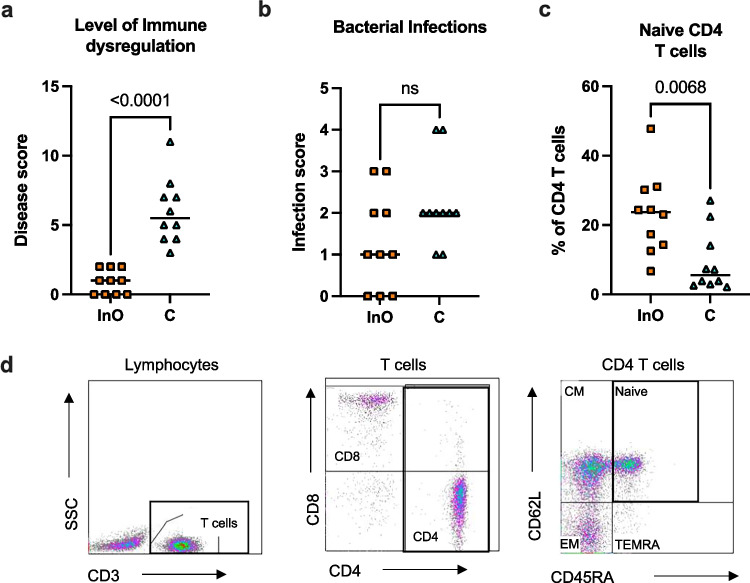
Clinical phenotype of CVID patients. The level of immune dysregulation was graded 0–4, for each of the following items; lymphocytic proliferation, autoimmunity, and gastroenteropathy, resulting in total disease scores of 0–12 (**a**). Infection score was graded 0–4, based on the average of bacterial infections per year (**b**). Frequencies of naïve (CD45RA^+^ CD62L^+^) CD4 T cells in peripheral blood. Reference values for naïve CD4 (10–90 percentiles), 30–70% of CD4 (**c**). Representative gating for naïve CD4 T cells. InO, CVID infection only; C, CVID with immune dysregulation complications; CM, central memory; EM, effector memory; TEMRA, terminally differentiated RA positive. The Mann–Whitney test was used for comparison between groups

CVID_C_ patients (with severe immune dysregulation) and CVID_InO_ patients (with infection-only phenotype) were included in the study (Table 1). All patients had ongoing immunoglobulin replacement therapy but had not received any immunomodulatory drugs at the time when the samples were collected. The disease-infectious scoring system used by us had a strong correlation with previously published [30] disease severity score for CVID and CVID-like disorders (Fig. S1). Information about mild manifestations such as uncomplicated airway infections and isolated elevation of alkaline phosphatase in infection-only patients were missing in our dataset and not included in the comparison. In the CVID_C_ group, disease scores, including polyclonal lymphoproliferation, autoimmunity, and gastroenteropathy, ranged between 3 and 11, compared to 0–2 in the CVID_InO_ group (Fig. 1a). There were no differences in the infection scores between the two groups (Fig. 1b). Even if the CD4 T cell counts were similar between the groups, the frequencies of peripheral naïve CD4 T cells were lower in CVID_C_ patients, further reflecting a more severe clinical phenotype (Fig. 1c–d). Seven of the CVID patients have been subjected to exome analysis targeting genetic variants in accordance with IUIS Phenotypical Classification for Human Inborn Errors of Immunity [14]. All genotyped patients belonged to the group with immune dysregulation, and one patient was diagnosed with CTLA4 haploinsufficiency. In two patients, heterozygous variants of uncertain significance have been detected involving a member of the VAV gene family and a gene coding for an immunoglobulin like receptor. No pathological variant was detected in any of the other tested patients. Two of the patients in the CVIDc group that have not been genotyped have mild immune dysregulation (scores 3–4), and the third is deceased with no family history of CVID, which may explain why gene testing has not been performed.

### Mapping to Small RNA Databases

Sequencing technology was used to measure the expression of small RNAs (15–55 bases) extracted from plasma. In total, 262 small RNAs were detected in all samples, and another 143 were found in more than 70% of the samples. Percentages of the reads that could be mapped to the miRBase or piRNA database varied between 6.4 and 39% across all three groups (Fig. S2).

### Shared and Unique Circulating miRNA Signatures in CVID Patients with Different Phenotypes

**Fig. 2:**
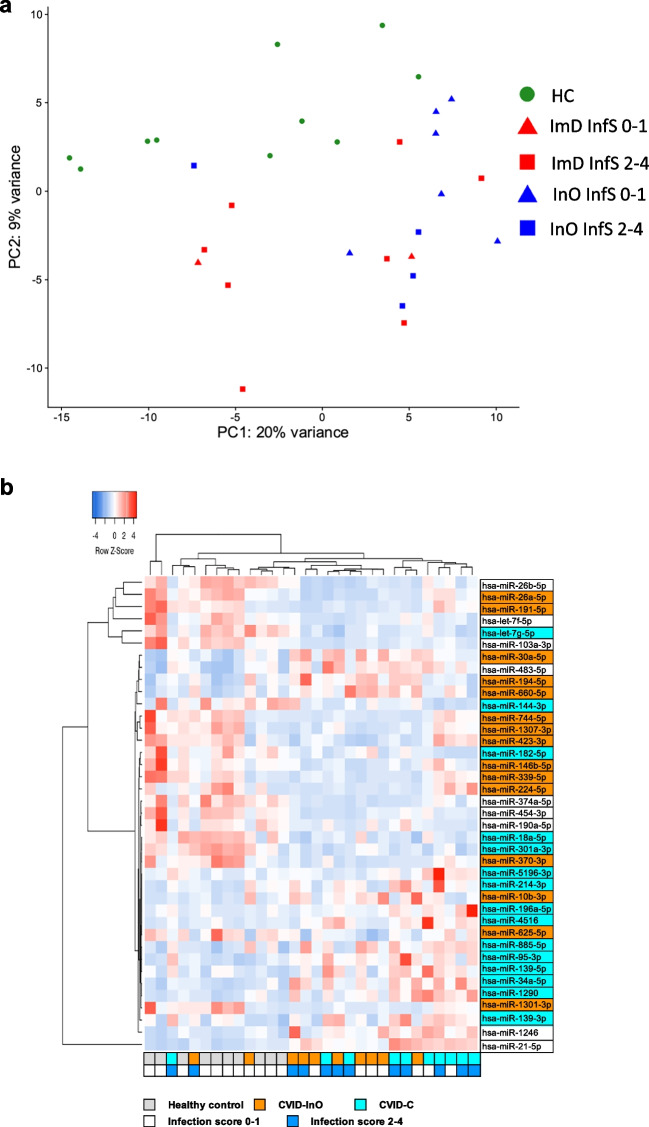
Differential circulating miRNA expression. Principal component analysis of 500 miRNAs with the highest variation. Green circles indicate healthy controls (HC), CVID with immune dysregulation complications (C) is indicated in red and CVID infection only (InO) in blue. Triangles indicate infection score (InfS) 0–1 and squares indicate infection score 2–4 (**a**). Heatmap of 39 deregulated miRNAs compared to HC, defined as FDR < 0.01 and log2FC > 1.1 or <  − 0.7. miRNAs in turquoise, CVID_C_ vs HC; in orange, CVID_InO_ vs HC; in white, overlap CVID_C_ and CVID_InO_ (**b**)

Dimensional reduction of transformed raw counts of the 500 miRNAs with the highest variance across samples showed a separation between healthy controls and CVID patients when the data were projected to principal component (PC) 1 and PC2 (Fig. 2a). CVID patients clustered into two groups, and one cluster was dominated by patients with an increased infection score, suggesting that the burden of infections may influence the plasma miRNA profiles. We performed a pair-wise comparison of the levels of circulating miRNAs between the CVID_C_ group (*n* = 10) and healthy controls (*n* = 10), and the CVID_InO_ group (*n* = 10) and healthy controls, to identify significantly deregulated miRNAs in plasma. When comparing CVID_C_ patients and healthy controls, a total of 24 circulating miRNAs were deregulated in CVID_C_ patients, when a false discovery rate (FDR) of < 0.01 was applied (Table S3a). Thirteen miRNAs were upregulated in CVID_C_, and another 11 were downregulated. When comparing CVID_InO_ patients and healthy controls, a total of 24 circulating miRNAs were deregulated (Table S3b). Seven miRNAs were upregulated, and another 17 were downregulated in the CVID_InO_ group when compared to healthy controls. The normalized expressed reads of the 39 significantly deregulated miRNAs were analyzed, and we found a clear separation between CVID patients and healthy controls and that the infection burden, *i.e.,* infection scores, did not contribute to the separation between the two CVID groups (Fig. 2b). ORA using the miRNA enrichment and analysis and annotation tool (miEAA) [31] identified 14 candidate genes (FDR < 0.01) targeted by the 24 miRNAs deregulated in CVID_C_ patients and eight candidate genes (FDR < 0.01) targeted in CVID_InO_ patients (Table 2). Five of the 14 candidate genes predicted by the CVID_C_ deregulated miRNAs were annotated with gene ontology (GO)-child terms of “immune system process.” Two of the most significantly affected genes, DAPK1 and WNT5A, were annotated with the GO-term “cellular response to type 2 interferons” (Table 2). Together, these findings indicate that miRNAs may modulate immune functions associated with CVID-related ImD.

**Table 2:** Predicted candidate gene targets biological process annotations

Target	FDR	Expected	Observed	Annotated BP GO-terms		
CVID with immune dysregulation vs HC				All^#^	ISP*	Biological process
CASP3	0.0017	0.3308	6	55	5	T cell homeostasis, B cell homeostasis
DAPK1	0.0017	0.1890	5	19	1	Cellular response to type II interferon
PTEN	0.0077	1.1343	8	91	4	T cell proliferation
BCL2	0.0097	0.633	6	130	11	T cell homeostasis, B cell homeostasis
TGFBR3	0.0097	0.6276	6	35	2	Immune response
NFKB1	0.0092	0.1607	4	32	0	
CKAP5	0.0021	0.0945	4	10	0	
ADNP	0.0078	0.0472	3	23	0	
JMY	0.0092	0.5671	6	9	0	
PDK4	0.0092	0.0567	3	16	0	
GGA3	0.0097	0.3497	5	10	0	
USP47	0.0097	0.1891	4	16	0	
DERL1	0.0097	0.0662	3	10	0	
PROSER1	0.0094	0.0656	3	N/A	-	
DICER1	0.0098	0.6617	6	20	0	
NUS1	0.0098	0.6617	6	11	0	
ZNF480	0.0098	0.1985	4	1	0	
CVID infection only				All^#^	ISP*	Biological process
WNT5A	0.0067	0.2930	5	153	6	Cellular response to type II interferon
PDE4B	0.0067	0.0473	3	15	4	Neutrophil homeostasis
SMN1	0.0041	0.0851	4	4	0	
BAZ1B	0.0067	0.1229	4	10	0	
SERPINB5	0.0067	0.1323	4	5	0	
STUB1	0.0067	0.0378	3	27	0	
TCEAL1	0.0099	0.1607	4	N/A	-	
RAI14	0.0099	0.0567	3	2	0	

*HC*, healthy controls; *FDR*, false discovery rate; ^*#*^*Total number annotated gene ontology (GO) terms* biological process (BP). *Number annotated BP GO-terms within the item immune system process (ISP)

### Differences in Circulating miRNA Signatures in CVIDC- and CVID_InO_ Patients

**Fig. 3:**
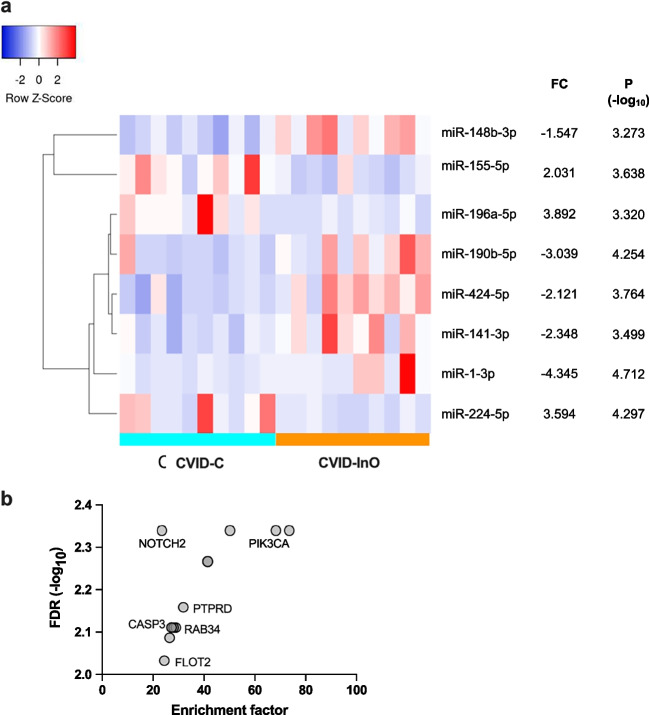
Differences in circulating miRNA signatures in CVID_C_ and CVID_InO_. Differently expressed miRNAs in CVID_C_ compared to CVID_InO_ (**a**). Predicted gene targets by differently expressed miRNAs over representation analysis (ORA). Labeled gene targets are annotated with gene ontology biological process item immune system process (**b**). Enrichment factor: number observed/number expected miRNAs. C, CVID with immune dysregulation; InO, CVID infection only; p, refers to Exact Test; FC, fold change; FDR, false discovery rate

When the two CVID groups were compared with each other, we applied FDR < 0.1. Three miRNAs were increased, and five miRNAs were decreased in CVID_C_ patients compared to CVID_InO_ patients (Fig. 3a). ORA identified 13 candidate genes (FDR < 0.01) targeted by the eight miRNAs deregulated in the CVID_C_-group compared to the CVID_InO_-group (Fig. 3b). Among the 13 candidate genes, NOTCH2, PIK3CA, PTPRD, CASP3, RAB34, and FLOT2 were annotated with GO terms related to the immune system process, including “T- and B cell homeostasis”. Except for CASP3, the predicted candidate genes were distinct from the predicted candidate genes when comparing CVID patients with healthy controls. In summary, the miRNA patterns differed between CVID_InO_- and CVID_C_ patients, suggesting distinct sets of predicted candidate genes.

### Targeted Analysis Deregulated miRNAs with Real-Time PCR

IFN-γ is a potential underlying driver of CVID-related ImD [10]. DAPK1 is a protein kinase, which regulates IFN-γ induced gene expression [32]. When, as the next step, we wanted to verify the plasma levels of the miRNAs sharing the predicted gene target DAPK1 with real-time qPCR. In addition, the two most deregulated miRNAs between the CVIDInO- and CVIDC group were analyzed by qPCR (Table S4). Eight miRNAs were analyzed by RT-PCR. Four of these miRNAs were only detected in 3–70% of the samples and were excluded from further analysis due to low call rate. One sample (CVIDInO) had an overall low call rate and was omitted from analysis. Of a total of eight analyzed miRNAs, four were detected in > 75% of samples and selected for further analysis (Table S4).

### Correlation of Circulating miRNAs with Plasma Levels of Inflammatory Proteins

**Fig. 4:**
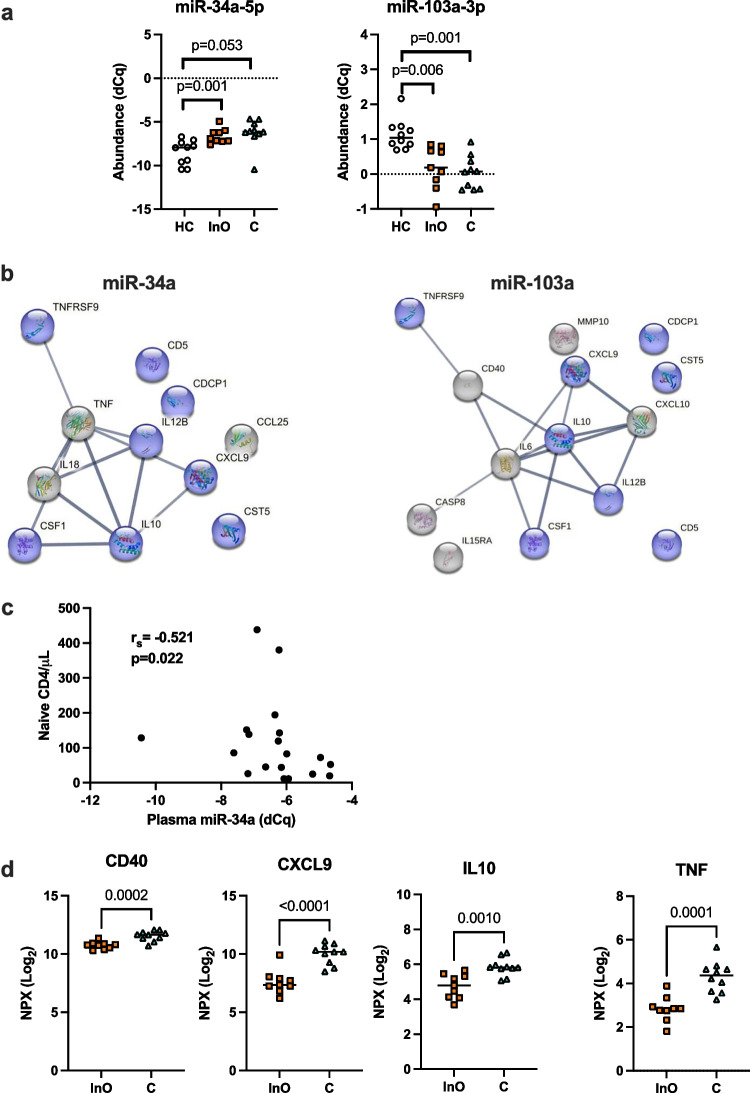
Correlation of circulating mir-34a-5p with inflammatory plasma protein and naïve CD4 T cell levels. Deregulated miRNAs detected by real-time qPCR in plasma (**a**). Predicted protein–protein interactions by the STRING database vs 11.5 of the inflammatory proteins correlating strongly with mir-34a-5p and mir-103a-3p, respectively. We used a high confidence (> 0.700) for the STRING analyses and the line thickness indicates the strength of the data support. Blue indicates factors shared by mir-34a-5p and mir-103-3p (**b**). Correlation of mir-34a-5p with naïve (CD45RA^+^ CD62L^+^) CD4 T cell numbers in CVID patients (**c**). Differences in plasma protein levels of putative mir-34a-5p targets between CVID groups (**d**). dCq, normalized cycle of quantification; HC, healthy controls; InO, CVID with infection only and CVID_C_, CVID with immune dysregulation complications. The Kruskal–Wallis test with Dunn correction was used for comparisons between multiple groups and a *t*-test for comparisons between two groups. r_s_ indicates Spearman correlation. Inflammatory factors are depicted by their gene names

We found a negative correlation between mir-34a-5p and mir-103a-3p normalized dCq plasma levels (Spearman correlation, *r*_s_ =  − 0.53 and *p* = 0.003), and a positive correlation between mir-103a-3p and mir-301a-3p (*r*_s_ = 0.469 and *p* = 0.001) dCq levels (Fig. S3). The plasma levels of mir-34a-5p were higher in CVID_InO_- and CVID_C_ patients compared to the controls, whereas the levels of mir-103a-3p were decreased in CVID_InO_- and CVID_C_ patients compared to healthy controls (Fig. 4a). When comparing CVID plasma inflammatory protein profiles, 32 factors were elevated (*T*-test, FDR < 0.05) compared to the healthy controls (Table 3). To outline any association of deregulated miRNAs, circulating dCq levels were correlated to normalized levels of 32 deregulated proteins in plasma. In total, 15 proteins showed a positive correlation (*r*_s_ ≥ 0.60 and *p* < 0.001) with mir-34a-5p or a negative correlation (*r*_s_ ≤ -0.60 and *p* < 0.001) with mir-103a-3p dCq levels (Table 3). No protein correlated with mir-301a-3p. Most of the proteins (*n* = 8) correlated with both mir-34a-5p and mir-103a-3p dCq levels: another three proteins correlated with mir-34a-5p and another six with mir-103a-3p (Fig. S4). We then used the proteins correlating with mir-34a-5p or mir-103a-3p to generate a protein interaction (PPI) network. Known and predicted interactions were revealed between seven of 11 factors correlating with mir-34a-5p and between eight of 14 factors correlating with mir-103a-3p (Fig. 4b). Proteins associated with mir-34a-5p or mir-103a-3p were predicted to be enriched (*p* = 4.3 × 10^−11^ and *p* = 6.5 × 10^−13^, respectively). TNFRSF9, CXCL9, IL10, IL12B, and CSF-1 were shared in the predicted networks of proteins correlating with mir-34a-5p and mir-103a-3p. Functional annotation predicted the biological process “Regulation of chronic inflammatory response to antigenic stimulus” (GO:0002874), involving IL10 and IL18, to be the interaction with the highest strength in the network, correlating with mir-34a-5p (strength 3.07 and FDR = 0.0012). For proteins correlating with mir-103-3p, the biological process “Negative regulation of inflammatory response to antigenic stimulus” (GO:0002862), involving IL10 and IL12B, had the highest strength (strength 2.33 and FDR = 0.0079). In summary, our results suggest that increased levels of mir-34a-5p and decreased levels of mir-103a-3p may contribute to the chronic inflammatory response in CVID-related ImD.

**Table 3:** Correlation of plasma protein levels with circulating miRNA dCq levels

Assay	CVID C(*n* = 10)		CVID InO (*n* = 8)		HC (*n* = 9)		mir-34a (*n* = 27)		mir-103a (*n* = 27)		mir-301a (*n* = 27)	
NPX (Log2)	Mean	SD	Mean	SD	Mean	SD	*r*	*p* (-log_10_)	*r*	*p* (-log_10_)	*r*	*p* (-log_10_)
IL-12B	7.52	0.46	6.16	0.78	5.05	0.39	0.71	4.63	− 0.65	3.69	− 0.26	0.76
TNFRSF9	8.87	0.52	7.19	0.48	6.27	0.19	0.68	4.17	− 0.63	3.51	− 0.33	1.06
TNF	4.30	0.64	2.83	0.52	2.20	0.35	0.67	4.02	− 0.58	2.90	− 0.22	0.59
IL10	5.85	0.45	4.75	0.62	3.19	0.12	0.66	3.93	− 0.62	3.38	− 0.41	1.52
CDCP1	4.47	0.78	2.44	0.65	1.93	0.32	0.66	3.87	− 0.70	4.50	− 0.31	0.98
CD5	6.44	0.47	4.93	0.35	4.37	0.16	0.66	3.86	− 0.67	4.09	− 0.21	0.56
CST5	5.38	0.46	4.98	0.49	4.60	0.24	0.66	3.83	− 0.70	4.40	− 0.28	0.83
CXCL9	10.00	0.79	7.61	0.92	6.48	0.36	0.65	3.70	− 0.71	4.60	− 0.28	0.84
IL18	9.85	0.85	8.41	0.59	7.66	0.29	0.64	3.57	− 0.52	2.35	− 0.19	0.47
CCL25	6.28	0.39	6.12	0.32	5.70	0.36	0.61	3.19	− 0.56	2.73	− 0.17	0.41
CSF-1	9.99	0.14	9.52	0.15	9.19	0.19	0.60	3.11	− 0.75	5.36	− 0.34	1.12
IL6	4.54	0.75	3.31	0.58	2.27	0.44	0.59	3.03	− 0.74	5.13	− 0.32	1.00
CCL3	6.09	0.65	4.62	0.53	4.02	0.46	0.58	2.90	− 0.53	2.45	− 0.18	0.45
CD244	7.24	0.38	6.40	0.22	6.15	0.36	0.58	2.87	− 0.57	2.79	− 0.13	0.28
IL-15RA	2.06	0.43	1.05	0.33	0.84	0.27	0.57	2.82	− 0.65	3.69	− 0.17	0.42
IFN-gamma	8.76	1.38	5.90	0.91	5.30	0.57	0.56	2.76	− 0.47	1.93	− 0.16	0.37
CXCL10	11.69	0.52	9.41	0.89	8.84	0.36	0.56	2.72	− 0.67	4.06	− 0.20	0.50
CD6	6.86	0.75	4.93	0.56	4.44	0.52	0.54	2.53	− 0.55	2.64	− 0.07	0.15
CD8A	11.62	0.65	10.49	0.72	9.98	0.50	0.54	2.52	− 0.45	1.80	0.00	0.01
CASP-8	1.45	0.27	1.05	0.32	0.64	0.18	0.53	2.40	− 0.71	4.65	− 0.27	0.76
CCL19	11.19	0.69	8.97	0.79	8.68	0.91	0.52	2.37	− 0.44	1.69	− 0.03	0.05
CXCL6	8.71	0.48	7.63	0.67	7.12	0.68	0.50	2.18	− 0.52	2.38	− 0.17	0.41
CD40	11.57	0.40	10.75	0.28	10.57	0.30	0.50	2.14	− 0.63	3.48	0.00	0.01
CCL20	7.64	0.79	6.53	1.15	5.96	0.82	0.48	2.01	− 0.33	1.07	0.14	0.32
LIF-R	3.80	0.31	3.21	0.26	3.13	0.17	0.47	1.91	− 0.46	1.88	− 0.33	1.07
TNFB	5.60	0.41	4.42	0.62	3.81	0.41	0.45	1.82	− 0.42	1.56	− 0.08	0.16
IL-18R1	8.87	0.47	7.84	0.49	7.66	0.36	0.44	1.72	− 0.54	2.55	− 0.11	0.24
PD-L1	6.85	0.37	5.85	0.33	5.72	0.34	0.43	1.68	− 0.57	2.77	− 0.20	0.52
CXCL11	9.63	0.94	7.30	0.58	7.41	0.61	0.43	1.66	− 0.47	1.91	0.04	0.08
TNFSF14	4.50	0.64	3.49	0.27	3.26	0.52	0.39	1.40	− 0.56	2.74	0.05	0.10
MMP-10	8.74	0.35	8.12	0.65	7.78	0.39	0.33	1.08	− 0.64	3.64	− 0.16	0.39
AXIN1	1.70	0.49	1.17	0.74	2.41	1.24	− 0.38	1.31	0.11	0.23	0.40	1.47

*dCq*, normalized cycle of quantification; *C*, CVID with immune dysregulation complications; *InO*, CVID infection only; *NPX*, normalized protein expression; *SD*, standard deviation; *r*, Spearman correlation

### Circulating mir-34a-5p Correlated with Naïve CD4 T Cells in CVID

Low levels of naïve CD4 T cells represent a finding strongly associated with CVID-related ImD without a direct connection to disease-causing genetic variants [7], which support that additional mechanisms may contribute to the heterogeneity of the clinical phenotype seen in CVID. This led us to investigate potential associations between deregulated miRNAs and naïve CD4 T cells in CVID patients. We found that mir-34a-5p dCq levels correlated negatively with numbers of circulating naïve CD4 T cells (Fig. 4c). No such correlation was found for mir-103a-3p (data not shown). We next used GeneTrail2 to identify the predicted target genes of mir-34a-5p annotated with the ancestral GO biological process term “Immune system process” (GO:0002376). The putative targets were predicted to be overrepresented in the pathway since 176 were detected compared to 113 expected (*p* = 2.0 × 10^−8^). We next compared the putative mir-34a-5p targets with the proteins analyzed in plasma and found that four putative mir-34a-5p targets (CD40, CXCL9, IL10, and TNF) overlapped (Table S5). The plasma levels of all four mir-34a-5p targets CD40, CXCL9, IL10, and TNF were all significantly higher in CVID_C_ patients compared to CVID_InO_ patients (Fig. 4d). Together these findings support a potential role of mir-34a-5p in CVID-related ImD.

## Discussion

This study is, to our knowledge, the first to delineate the circulating miRNAome in CVID and to correlate the findings to CVID-related ImD. We found various miRNAs that distinguished the CVID patients with severe ImD complications (CVID_C_) from the infection-only CVID phenotype (CVID_InO_), as well as the CVID groups from healthy controls. The burden of non-infectious complications in CVID was assessed by a clinical severity scoring system assessing lymphoproliferation, autoimmunity, and gastrointestinal complications [10]. The accuracy of the scoring system was validated using the more extensive CVID disease severity score published by Ameratunga in 2018 [30]. Incomplete data of mild manifestations should not affect the results significantly since mild manifestations only generated a score of 1 using the Ameratunga scoring system. Twenty-four miRNAs were altered between CVID_C_ patients and the controls, and 24 between CVID_InO_ patients and the controls. Several genes involved in adaptive immune mechanisms, including a gene involved in type 2 interferon signaling, were predicted candidate gene targets of deregulated miRNAs in the CVID_C_ group. We and others have previously shown that IFN-γ correlates with CVID-related ImD [10, 32]. The top predicted gene targets in the CVID_InO_ group were merely associated with inflammatory processes and innate immunity. Low naïve CD4 T cells are associated with severe disease and a poor outcome in CVID. Targeted analysis with qPCR showed a negative correlation between naïve CD4 T cell numbers and mir-34a-5p expression, and the plasma levels of four putative mir-34a-5p gene targets, *i.e.*, CD40, CXCL9, IL10, and TNF, were elevated in CVID_C_ compared to CVID_InO_ patients. Determining correlations between miRNAs and clinical and laboratory findings may provide new mechanistic insights into CVID-related ImD since circulating miRNA transcripts reflect intracellular gene network activity in tissues [26].

The majority of CVID cases are sporadic and the onset can occur at any age. A few pathogenic gene variants have been associated with CVID [14, 15]. However, only a minority of CVID cases appear to be the result of a monogenic variant. Instead, transcriptional and post-transcriptional modifications may contribute to the clinical penetrance of genetic variants in CVID, alone or in combination with other triggers such as infections [16]. Increasing evidence indicates that miRNAs play a key role in biological processes by their modulation of gene expression [33]. While the plasma miRNA profiles clearly discriminated between CVID patients and healthy controls, the differences between the CVID_C_- and the CVID_InO_ patients were less pronounced. However, the distinct circulating miRNA profiles found in CVID_C_- and in CVID_InO_ patients compared to the controls suggest that deregulated expression of circulating miRNAs may contribute to the heterogeneity of CVID phenotypes. In addition, ORA of differently expressed miRNAs identified several potential gene targets involved in T- and B cell homeostasis in CVID patients with severe ImD. These findings imply that miRNAs may contribute to the changes in T- and B-cell populations found in this group of CVID. For instance, DAPK-1 was one of the most highly predicted gene targets in the CVID_C_ group compared to the controls. DAPK-1 is a tumor suppressor induced by IFN-γ [34, 35] and can also influence the expression of IFN-γ [36]. Altered DAPK1 activity may impair CVID T cell functionality, seeing that DAPK1 has been linked to the regulation of T cell function [37, 38]. WNT5A was another predicted target when comparing the miRNA profiles of CVID_InO_ patients with healthy controls. WNT5A, like DAPK1, is a tumor suppressor which can also modulate T cell activation [39–41]. Taken together, dysregulation of DAPK1 and WNT5A may contribute to the heterogeneity of the immune defects observed in CVID.

When comparing the plasma miRNA profiles of the two CVID groups, the highest upregulated miRNA in CVID_C_ was mir-224-5p. Mir-224 expression is upregulated via NFΚB and has been reported to attenuate the inflammatory responses by interaction with the acute phase protein pentraxin 3 [42, 43]. The CVID_C_ group had low levels of mir-1-3p, and this miRNA has been found to modulate immune cell functions and contribute to inflammatory damage in an experimental model of sepsis [44, 45]. Increased expression of miR-224-5p and reduced expression of miR-1-3p may regulate the inflammatory response in CVID related ImD. Bioinformatic analysis of deregulated miRNA in the CVID_C_ group compared to the CVID_InO_ group identified several putative gene targets such as NOTCH2, which is important for T cell responses [46], the PIK3CA, part of the PI3K signaling pathway [47], and the inflammatory caspase 3, a key protein in apoptosis and pyroptosis pathways [48]. In summary, deregulated miRNA in CVID may potentially modulate fundamental cellular processes and thereby influence CVID-related ImD.

MiRNA raw count means, significant fold change between the CVID_C_- and CVID_InO_ groups, and putative involvement in IFN-γ signaling were factors considered in the selection of miRNAs for the validation assay with real-time PCR. Among the miRNAs in the validation assay, we found correlating levels of mir-103a-3p with mir-34a-5p and mir-301a-3p. In addition, both mir-103a-3p and mir-34a-5p correlated with inflammatory plasma proteins such as CXCL9, IL10, and IL12B. Protein–protein-interaction (PPI) network analysis indicated proteins regulating immune responses to be overrepresented in the mir-103a-3p cluster and the mir-34a-5p clusters. Experimental models have suggested that mir-103a-3p both promotes and ameliorates inflammatory responses [49, 50] and that this miRNA is upregulated in rheumatoid arthritis and connected to disease activity [51]. We found a negative correlation of mir-103a-3p with inflammatory proteins, which may be indicative of an anti-inflammatory activity in CVID. It is unlikely that mir-103a-3p specifically counteracts IFN-γ driven inflammation since plasma levels were similar in the two CVID groups. The net effect of mir-103a-3p in CVID remains to be determined.

Increased levels of mir-34a-5p have previously been reported in the whole blood of lung cancer patients [52] and in the plasma of people living with HIV, even after prolonged control of viral replication [53]. Functional in vitro studies have shown that mir-34a-5p modulates T cell signaling and the expression of IFN-γ induced CXCR3 ligands such as CXCL9, CXCL10, and CXCL11, which are chemoattractants for activated T cells; hence, mir-34a-5p may act as a major hub of T cell regulatory networks [54–56]. These findings support a role for mir-34a-5p in the pathogenesis of CVID-related ImD, which is associated with upregulation of the Tbet-CXCR3-axis with increased numbers of T-helper 1 skewed T cells and increased IFN-γ-production [10, 32]. Moreover, PPI analysis of plasma proteins correlating with mir-34a-5p predicted the enrichment of factors involved in a chronic inflammatory response, and the highest levels of mir-34a-5p were found in CVID_C_ patients, which may also imply a role of mir-34a-5p in CVID related ImD. Among the predicted immune system process gene targets of mir-34a-5p, we analyzed the plasma levels of four gene products (CD40, CXCL9, IL10, and TNF). They were all increased in CVID_C_ patients compared to CVID_InO_ patients and this may reflect upregulated mir-34a-5p activity. CXCL9 and TNF are Th1 cytokines. Mir-34a-5p is considered a tumor suppressor acting by augmenting Th1 T cell responses and preventing cancer cell immune checkpoint upregulation and subsequent immune evasion [57, 58]. Hence, it can be hypothesized that mir-34a-5p contributes to the enhanced CD4 T cell differentiation in CVID-related ImD, which is supported by negative correlations of naïve CD4 T cells and mir-34a-5p levels in this study. If the significance of mir-34a-5p in CVID-related ImD can be verified in functional studies, blocking of mir-34a-5p may potentially be a new therapeutic approach to consider.

Our study has some limitations. First is the restricted number of patients included and their heterogeneity when it comes to the clinical manifestations of CVID. Despite this, we identified differences in miRNA profiles when comparing CVID patients with- and without severe ImD that predicted enrichment of target genes involved in IFN-γ signaling. Furthermore, the distribution of male vs female in the infection only group was highly skewed towards male, which is a limitation, but when exploring the whole cohort, we did not find any significant differences in dCq-levels of miRNAs between male and female participants, so it should have a limited impact on the study. Moreover, it is an explorative study, and the findings need to be validated by longitudinally analyzing a larger study cohort, and an alternative study design is required to dissect the potential mechanistic role of miRNAs in the development of CVID-related ImD.

## Conclusions

In conclusion, we identified several circulating miRNAs that were associated with CVID-related ImD, which were predicted to target genes involved in IFN-γ-driven inflammation. Levels of mir-34a correlated with inflammatory factors and decreased numbers of naïve CD4 T cells. Collectively, our data show that miRNAs correlate with the inflammatory response in CVID and may help us understand the pathogenesis of CVID-related ImD. However, further studies are needed to elucidate the role of miRNAs in the development of CVID-related ImD.

### Supplementary Information

Below is the link to the electronic supplementary material.

Supplementary file1 (PDF 237 KB)

Supplementary file2 (PDF 109 KB)

Supplementary file3 (XLSX 23 KB)

## Data Availability

The datasets generated during the current study are available from the corresponding author on reasonable request.

## References

[1] Ameratunga R, Brewerton M, Slade C, Jordan A, Gillis D, Steele R (2014). Comparison of diagnostic criteria for common variable immunodeficiency disorder. Front Immunol.

[2] Chapel H, Lucas M, Lee M, Bjorkander J, Webster D, Grimbacher B (2008). Common variable immunodeficiency disorders: division into distinct clinical phenotypes. Blood.

[3] Cunningham-Rundles C, Bodian C (1999). Common variable immunodeficiency: clinical and immunological features of 248 patients. Clin Immunol (Orlando, Fla).

[4] Resnick ES, Moshier EL, Godbold JH, Cunningham-Rundles C (2012). Morbidity and mortality in common variable immune deficiency over 4 decades. Blood.

[5] Malphettes M, Gerard L, Carmagnat M, Mouillot G, Vince N, Boutboul D (2009). Late-onset combined immune deficiency: a subset of common variable immunodeficiency with severe T cell defect. Clin Infect Dis: Off Publ Infect Dis Soc Am.

[6] Mouillot G, Carmagnat M, Gerard L, Garnier JL, Fieschi C, Vince N (2010). B-cell and T-cell phenotypes in CVID patients correlate with the clinical phenotype of the disease. J Clin Immunol.

[7] von Spee-Mayer C, Koemm V, Wehr C, Goldacker S, Kindle G, Bulashevska A (2019). Evaluating laboratory criteria for combined immunodeficiency in adult patients diagnosed with common variable immunodeficiency. Clin Immunol (Orlando, Fla).

[8] Berbers RM, Drylewicz J, Ellerbroek PM, van Montfrans JM, Dalm V, van Hagen PM (2021). Targeted proteomics reveals inflammatory pathways that classify immune dysregulation in common variable immunodeficiency. J Clin Immunol.

[9] Cols M, Rahman A, Maglione PJ, Garcia-Carmona Y, Simchoni N, Ko HM (2016). Expansion of inflammatory innate lymphoid cells in patients with common variable immune deficiency. J Allergy Clin Immunol.

[10] Hultberg J, Ernerudh J, Larsson M, Nilsdotter-Augustinsson Å, Nyström S (2020). Plasma protein profiling reflects T(H)1-driven immune dysregulation in common variable immunodeficiency. J Allergy Clin Immunol.

[11] Bethune C, Egner W, Garcez T, Huissoon A, Jolles S, Karim Y (2019). British Society for Immunology/United Kingdom Primary Immunodeficiency Network consensus statement on managing non-infectious complications of common variable immunodeficiency disorders. Clin Exp Immunol.

[12] Hurst JR, Verma N, Lowe D, Baxendale HE, Jolles S, Kelleher P (2017). British Lung Foundation/United Kingdom Primary Immunodeficiency Network consensus statement on the definition, diagnosis, and management of granulomatous-lymphocytic interstitial lung disease in common variable immunodeficiency disorders. J Allergy Clin Immunol Pract.

[13] Abolhassani H, Hammarström L, Cunningham-Rundles C (2020). Current genetic landscape in common variable immune deficiency. Blood.

[14] Bousfiha A, Moundir A, Tangye SG, Picard C, Jeddane L, Al-Herz W (2022). The 2022 update of IUIS phenotypical classification for human inborn errors of immunity. J Clin Immunol.

[15] Edwards ESJ, Bosco JJ, Ojaimi S, O'Hehir RE, van Zelm MC (2021). Beyond monogenetic rare variants: tackling the low rate of genetic diagnoses in predominantly antibody deficiency. Cell Mol Immunol.

[16] Ramirez NJ, Posadas-Cantera S, Caballero-Oteyza A, Camacho-Ordonez N, Grimbacher B (2021). There is no gene for CVID - novel monogenetic causes for primary antibody deficiency. Curr Opin Immunol.

[17] Campos-Sanchez E, Martínez-Cano J, Del Pino ML, López-Granados E, Cobaleda C (2019). Epigenetic deregulation in human primary immunodeficiencies. Trends Immunol.

[18] Orange JS, Glessner JT, Resnick E, Sullivan KE, Lucas M, Ferry B (2011). Genome-wide association identifies diverse causes of common variable immunodeficiency. J Allergy Clin Immunol.

[19] Rodríguez-Ubreva J, Arutyunyan A, Bonder MJ, Del Pino-Molina L, Clark SJ, de la Calle-Fabregat C (2022). Single-cell Atlas of common variable immunodeficiency shows germinal center-associated epigenetic dysregulation in B-cell responses. Nat Commun.

[20] Moroney JB, Chupp DP, Xu Z, Zan H, Casali P (2020). Epigenetics of the antibody and autoantibody response. Curr Opin Immunol.

[21] Tsai DY, Hung KH, Chang CW, Lin KI (2019). Regulatory mechanisms of B cell responses and the implication in B cell-related diseases. J Biomed Sci.

[22] Wu H, Deng Y, Feng Y, Long D, Ma K, Wang X (2018). Epigenetic regulation in B-cell maturation and its dysregulation in autoimmunity. Cell Mol Immunol.

[23] Zhang J, Jima DD, Jacobs C, Fischer R, Gottwein E, Huang G (2009). Patterns of microRNA expression characterize stages of human B-cell differentiation. Blood.

[24] Babaha F, Yazdani R, Shahkarami S, Esfahani ZH, Abolhahassani H, Sadr M (2021). Evaluation of miR-210 expression in common variable immunodeficiency: patients with unsolved genetic defect. Allergol Immunopathol (Madr).

[25] Saccon TD, Dhahbi JM, Schneider A, Nunez Lopez YO, Qasem A, Cavalcante MB (2022). Plasma miRNA profile of Crohn’s Disease and Rheumatoid Arthritis Patients. Biology (Basel).

[26] Sohel MMH (2020). Circulating microRNAs as biomarkers in cancer diagnosis. Life Sci.

[27] Wilson JC, Kealy D, James SR, Plowman T, Newling K, Jagger C (2022). Integrated miRNA/cytokine/chemokine profiling reveals severity-associated step changes and principal correlates of fatality in COVID-19. iScience.

[28] Warnatz K, Denz A, Drager R, Braun M, Groth C, Wolff-Vorbeck G (2002). Severe deficiency of switched memory B cells (CD27(+)IgM(-)IgD(-)) in subgroups of patients with common variable immunodeficiency: a new approach to classify a heterogeneous disease. Blood.

[29] Szklarczyk D, Gable AL, Nastou KC, Lyon D, Kirsch R, Pyysalo S (2021). The STRING database in 2021: customizable protein-protein networks, and functional characterization of user-uploaded gene/measurement sets. Nucleic Acids Res.

[30] Ameratunga R (2018). Assessing disease severity in common variable immunodeficiency disorders (CVID) and CVID-like disorders. Front Immunol.

[31] Kern F, Fehlmann T, Solomon J, Schwed L, Grammes N, Backes C (2020). miEAA 2.0: integrating multi-species microRNA enrichment analysis and workflow management systems. Nucleic Acids Res.

[32] Unger S, Seidl M, van Schouwenburg P, Rakhmanov M, Bulashevska A, Frede N (2018). The TH1 phenotype of follicular helper T cells indicates an IFN-gamma-associated immune dysregulation in patients with CD21low common variable immunodeficiency. J Allergy Clin Immunol.

[33] Yao Q, Chen Y, Zhou X (2019). The roles of microRNAs in epigenetic regulation. Curr Opin Chem Biol.

[34] Gade P, Ramachandran G, Maachani UB, Rizzo MA, Okada T, Prywes R, et al. An IFN-γ-stimulated ATF6-C/EBP-β-signaling pathway critical for the expression of death associated protein Kinase 1 and induction of autophagy. Proc Natl Acad Sci U S A. 2012;109(26):10316-21. 10.1073/pnas.1119273109 PMC3387052 22699507

[35] Wang Q, Lin Y, Zhong W, Jiang Y, Lin Y (2021). Regulatory non-coding RNAs for death associated protein kinase family. Front Mol Biosci.

[36] Mukhopadhyay R, Ray PS, Arif A, Brady AK, Kinter M, Fox PL (2008). DAPK-ZIPK-L13a axis constitutes a negative-feedback module regulating inflammatory gene expression. Mol Cell.

[37] Wei Z, Du Q, Li P, Liu H, Xia M, Chen Y (2021). Death-associated protein kinase 1 (DAPK1) controls CD8(+) T cell activation, trafficking, and antitumor activity. Faseb J.

[38] Wei Z, Li P, He R, Liu H, Liu N, Xia Y (2021). DAPK1 (death associated protein kinase 1) mediates mTORC1 activation and antiviral activities in CD8(+) T cells. Cell Mol Immunol.

[39] Kremenevskaja N, von Wasielewski R, Rao AS, Schöfl C, Andersson T, Brabant G (2005). Wnt-5a has tumor suppressor activity in thyroid carcinoma. Oncogene.

[40] Kumawat K, Gosens R (2016). WNT-5A: signaling and functions in health and disease. Cell Mol Life Sci.

[41] Zhao F, Xiao C, Evans KS, Theivanthiran T, DeVito N, Holtzhausen A (2018). Paracrine Wnt5a-β-catenin signaling triggers a metabolic program that drives dendritic cell tolerization. Immunity.

[42] Rudnicki A, Shivatzki S, Beyer LA, Takada Y, Raphael Y, Avraham KB (2014). microRNA-224 regulates Pentraxin 3, a component of the humoral arm of innate immunity, in inner ear inflammation. Hum Mol Genet.

[43] Scisciani C, Vossio S, Guerrieri F, Schinzari V, De Iaco R, D'Onorio de Meo P, et al. Transcriptional regulation of miR-224 upregulated in human HCCs by NFκB inflammatory pathways. J Hepatol. 2012;56(4):855–61. 10.1016/j.jhep.2011.11.017 22178270

[44] Chen YL, Xie YJ, Liu ZM, Chen WB, Zhang R, Ye HX (2022). Omega-3 fatty acids impair miR-1-3p-dependent Notch3 down-regulation and alleviate sepsis-induced intestinal injury. Mol Med.

[45] Safa A, Bahroudi Z, Shoorei H, Majidpoor J, Abak A, Taheri M (2020). miR-1: A comprehensive review of its role in normal development and diverse disorders. Biomed Pharmacother.

[46] Tsukumo SI, Yasutomo K (2018). Regulation of CD8(+) T cells and antitumor immunity by notch signaling. Front Immunol.

[47] Ogino S, Galon J, Fuchs CS, Dranoff G (2011). Cancer immunology–analysis of host and tumor factors for personalized medicine. Nat Rev Clin Oncol.

[48] Jiang M, Qi L, Li L, Li Y (2020). The caspase-3/GSDME signal pathway as a switch between apoptosis and pyroptosis in cancer. Cell Death Discov.

[49] Lu Q, Ma Z, Ding Y, Bedarida T, Chen L, Xie Z (2019). Circulating miR-103a-3p contributes to angiotensin II-induced renal inflammation and fibrosis via a SNRK/NF-κB/p65 regulatory axis. Nat Commun.

[50] Zhou YP, Xia Q (2020). Inhibition of miR-103a-3p suppresses lipopolysaccharide-induced sepsis and liver injury by regulating FBXW7 expression. Cell Biol Int.

[51] Bagheri-Hosseinabadi Z, Mirzaei MR, Hajizadeh MR, Asadi F, Rezaeian M, Abbasifard M (2021). Plasma microRNAs (miR-146a, miR-103a, and miR-155) as potential biomarkers for rheumatoid arthritis (RA) and disease activity in Iranian patients. Mediterr J Rheumatol.

[52] Leidinger P, Backes C, Dahmke IN, Galata V, Huwer H, Stehle I (2014). What makes a blood cell based miRNA expression pattern disease specific?–A miRNome analysis of blood cell subsets in lung cancer patients and healthy controls. Oncotarget.

[53] Cuesta-Sancho S, Márquez-Ruiz D, Illanes-Álvarez F, Campaña-Gómez I, Martín-Aspas A, Trujillo-Soto MT (2023). Expression profile of microRNAs related with viral infectivity, inflammatory response, and immune activation in people living with HIV. Front Microbiol.

[54] Hart M, Nickl L, Walch-Rueckheim B, Krammes L, Rheinheimer S, Diener C (2020). Wrinkle in the plan: miR-34a-5p impacts chemokine signaling by modulating CXCL10/CXCL11/CXCR3-axis in CD4(+), CD8(+) T cells, and M1 macrophages. J Immunother Cancer.

[55] Hart M, Walch-Rückheim B, Friedmann KS, Rheinheimer S, Tänzer T, Glombitza B (2019). miR-34a: a new player in the regulation of T cell function by modulation of NF-κB signaling. Cell Death Dis.

[56] Lu C, Zhang X, Luo Y, Huang J, Yu M (2022). Identification of CXCL10 and CXCL11 as the candidate genes involving the development of colitis-associated colorectal cancer. Front Genet.

[57] Li WJ, Wang Y, Liu R, Kasinski AL, Shen H, Slack FJ (2021). MicroRNA-34a: potent tumor suppressor, cancer stem cell inhibitor, and potential anticancer therapeutic. Front Cell Dev Biol.

[58] Sallman DA, McLemore AF, Aldrich AL, Komrokji RS, McGraw KL, Dhawan A (2020). TP53 mutations in myelodysplastic syndromes and secondary AML confer an immunosuppressive phenotype. Blood.

