# Supplementary material for: Plasma Levels of mir-34a-5p Correlate with Systemic Inflammation and Low Naïve CD4 T Cells in Common Variable Immunodeficiency
Source: J Clin Immunol. 2023 Dec 22;44(1):21. doi: 10.1007/s10875-023-01618-0 (PMC10739380; doi:10.1007/s10875-023-01618-0)
Supplement: Supplementary file 1 — Supplementary file1 (PDF 237 KB) [file 10875_2023_1618_MOESM1_ESM.pdf]

Supplementary figures 1-4

Plasma levels of mir-34a-5p correlate with systemic inflammation and low naïve CD4 T cells in common variable immunodeficiency

Sofia Nyström[1,2]*, Jonas Hultberg[2], Emelie Blixt[1], Åsa Nilsdotter-Augustinsson[3] and Marie Larsson[2]
[1]Clinical Immunology and Transfusion Medicine, Department of Biomedical and Clinical Sciences, Linköping University, Linköping, Sweden
[2]Division of molecular medicine and virology, Department of Biomedical and Clinical Sciences, Linköping University, Linköping, Sweden
[3]Division of inflammation and infections, Department of Biomedical and Clinical Sciences, Linköping University, Linköping, Sweden
*Corresponding author: sofia.c.nystrom@liu.se

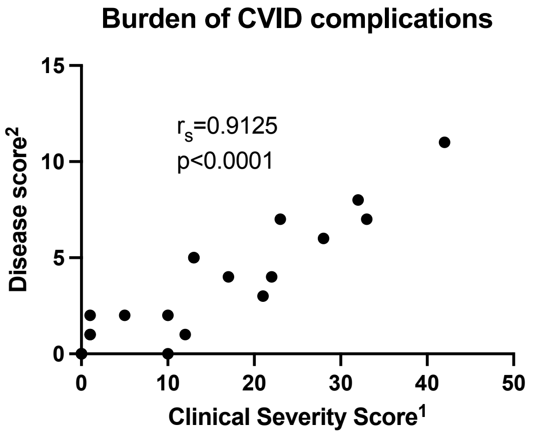

Fig S1. Validation of the CVID disease scoring system. Correlations of the CVID-related immune dysregulation scoring system used in this study (2) with an independent, more extensive CVID disease scoring system introduced by Ameratunga in 2018 (1).

1. **Ameratunga R**. Assessing Disease Severity in Common Variable Immunodeficiency Disorders (CVID) and CVID-Like Disorders. Front Immunol. 2018 Sep 28;9:2130. doi: 10.3389/fimmu.2018.02130. PMID: 30323807; PMCID: PMC6172311.
2. **Hultberg J**, Ernerudh J, Larsson M, Nilsdotter-Augustinsson Å, Nyström S. Plasma protein profiling reflects TH1-driven immune dysregulation in common variable immunodeficiency. J Allergy Clin Immunol. 2020 Aug;146(2):417-428. doi: 10.1016/j.jaci.2020.01.046. Epub 2020 Feb 11. PMID: 32057767.

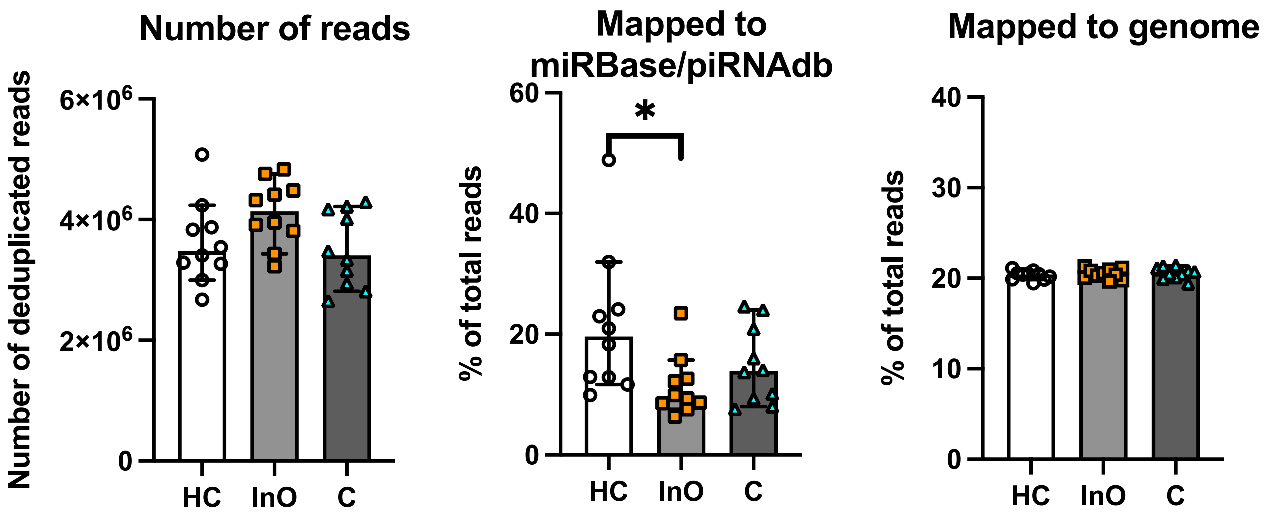

Fig. S2 miRNA mapping statistics and plasma miRNA pattern. Number of deduplicated reads and percentages of reads that were mapped to miRBase/piRNAdb and unannotated reads that were mapped to the genome. Kruskal-Wallis test was used for comparisons between groups. HC, helathy controls; InO, CVID infection-only; C, CVID with immune dysregulation complications. *Indicates $p<0.05$ Kruskal-Wallis test with Dunn correction.

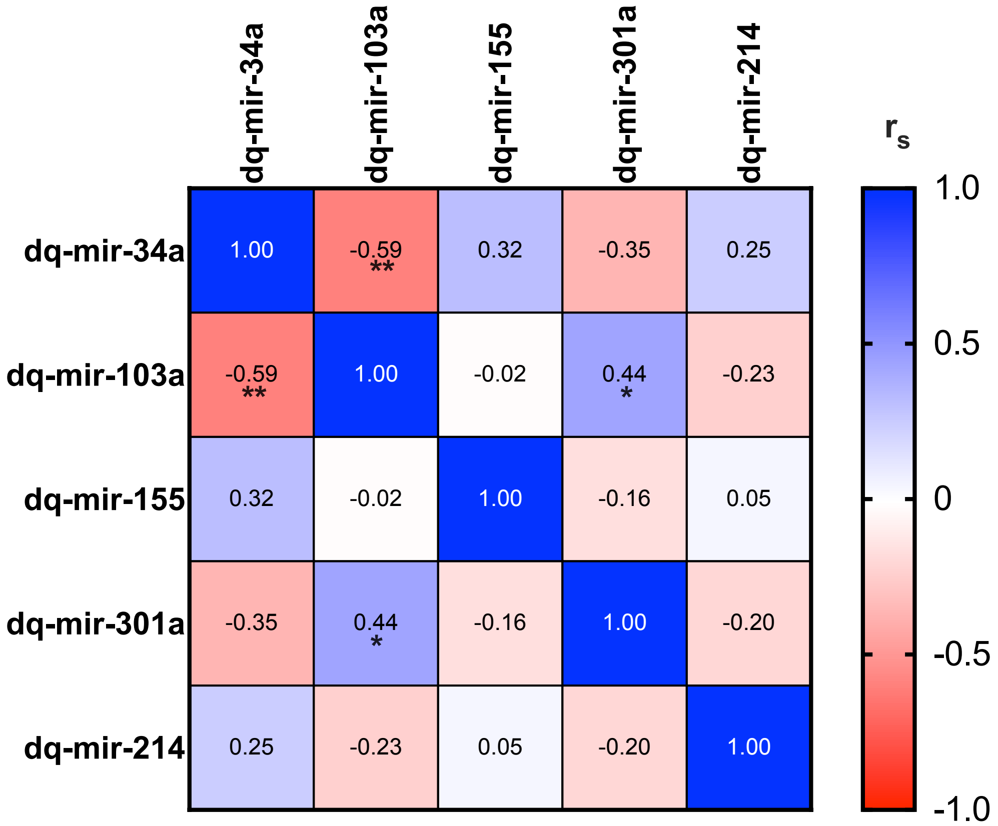

Fig. S3 Correlation of miRNA dCq plasma levels. Spearman correlation matrix of deregulated mir dCq levels show negative correlation between miR-103a and miR-34a and positive correlation between miR-103a and miR-301a. r_s, Spearman correlation; *p<0.05; **p<0.01.

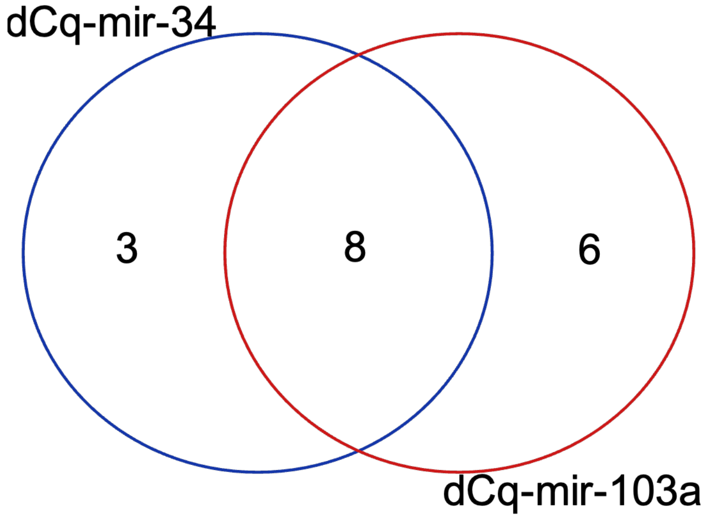

Fig. S4 Overlap of plasma protein correlating with miRNAs. Venn diagram of overlap between plasma inflammatory proteins which correlated positively ($r \geq 0.60$, $p<0.001$) with mir-34a dCq levels and negative ($r \leq -0.60$, $p<0.001$) with mir-103a dCq levels.
