# Supplementary material for: Plasma Levels of mir-34a-5p Correlate with Systemic Inflammation and Low Naïve CD4 T Cells in Common Variable Immunodeficiency
Source: J Clin Immunol. 2023 Dec 22;44(1):21. doi: 10.1007/s10875-023-01618-0 (PMC10739380; doi:10.1007/s10875-023-01618-0)
Supplement: Supplementary file 2 — Supplementary file2 (PDF 109 KB) [file 10875_2023_1618_MOESM2_ESM.pdf]

Supplementary Table S1

Plasma levels of mir-34a-5p correlate with systemic inflammation and low naïve CD4 T cells in common variable immunodeficiency

Sofia Nyström[1,2]*, Jonas Hultberg[2], Emelie Blixt[1], Åsa Nilsdotter-Augustinsson[3] and Marie Larsson[2]

[1]Clinical Immunology and Transfusion Medicine, Department of Biomedical and Clinical Sciences, Linköping University, Linköping, Sweden, [2]Division of molecular medicine and virology, Department of Biomedical and Clinical Sciences, Linköping University, Linköping, Sweden, [3]Division of inflammation and infections, Department of Biomedical and Clinical Sciences, Linköping University, Linköping, Sweden

*Corresponding author: sofia.c.nystrom@liu.se

Table S1 Key Resources

Reagent or Resource	Source	Identifier
**Biological samples**		
Human blood samples (CVID)	Linköping University Hospital, Sweden	
Human blood samples (Healthy donors)	Linköping University Hospital, Sweden	
**Reagents for flow cytometry**		
BD Multitest™ reagent CD3/CD8/CD45/CD4 with trucount tubes	https://www.bdbiosciences.com	#342447
BD FACS™ lysing solution	https://www.bdbiosciences.com	#349202

CD45RA-FITC clone L48	https://www.bdbiosciences.com	#335039
CD62L-APC clone Dreg56	https://www.bdbiosciences.com	#559772
CD4-PE-Cy7 clone SK3	https://www.bdbiosciences.com	#557852
CD8-APC-H7 clone SK1	https://www.bdbiosciences.com	#560179
CD3-Horizon V450 clone UCHT1	https://www.bdbiosciences.com	#560365
**Critical Commercial Reagents/Assays**		
miRNeasy kit	https://www.qiagen.com/	#217184
QIAseq miRNA Library Kit	https://www.qiagen.com/	#331502
miRCURY LNA RT Kit	https://www.qiagen.com/	#339340
Olink Target 96 Inflammation panel	https://olink.com/	vs3021
**Deposited Data**		
	This paper	GEO
**Software and Algorithms**		
Kaluza (software)	https://www.beckman.com	v2.1
Prism (software)	https://www.graphpad.com	v9.5.0
Qiagen CLC Genomics Server	https://digitalinsights.qiagen.com	v21.0.4<br>QIAseq miRNA quantification
Qiagen CLC Genomics Server	https://digitalinsights.qiagen.com	v21.0.4<br>RNA-Seq analysis

Qiagen CLC Genomics Workbench	https://digitalinsights.qiagen.com	v21.0.4<br><br>Empirical analysis of DGE
DSeq2	https://bioconductor.org	v1.28.1
Heatmapper	http://www.heatmapper.ca/	Expression
Calculate and draw custom Venn diagrams	https://bioinformatics.psb.ugent.be/webtools/Venn/	
miRNA Enrichment Analysis and Annotation Tool (miEAA)	https://ccb-compute2.cs.uni-saarland.de/mieaa2/	v2.0
Uniprot	https://www.uniprot.org/	Release 2022_05
STRING: Protein-Protein Interaction Networks Functional Enrichment Analysis	https://string-db.org/	Version 11.5
GeneTrail Advanced high-throughput enrichment analysis	https://genetrail.bioinf.uni-sb.de	Version 3.2
